# Imaging Outcomes of Emergency MRI in Patients with Suspected Cerebral Venous Sinus Thrombosis: A Retrospective Cohort Study

**DOI:** 10.3390/diagnostics13122052

**Published:** 2023-06-14

**Authors:** Tatu Happonen, Mikko Nyman, Pauli Ylikotila, Ville Kytö, Dan Laukka, Kimmo Mattila, Jussi Hirvonen

**Affiliations:** 1 Department of Radiology, Turku University Hospital, University of Turku, 20521 Turku, Finland; tjhapp@utu.fi (T.H.); mikko.nyman@varha.fi (M.N.); kimmo.mattila@varha.fi (K.M.); 2 Neurocenter, Turku University Hospital, University of Turku, 20521 Turku, Finland; pauli.ylikotila@varha.fi; 3 Heart Center, Clinical Research Center, Turku University Hospital, University of Turku, 20521 Turku, Finland; ville.kyto@varha.fi; 4 Department of Neurosurgery, Turku University Hospital, 20521 Turku, Finland; dan.laukka@varha.fi; 5 Department of Radiology, Tampere University Hospital, Tampere University, 33100 Tampere, Finland

**Keywords:** magnetic resonance imaging, emergency imaging, headache, thrombosis, cerebral venous sinus thrombosis

## Abstract

Cerebral venous sinus thrombosis (CVST) is a rare neurological emergency condition with non-specific symptoms. Imaging options to rule out CVST are computed tomography (CT) and magnetic resonance imaging (MRI). This study aimed to determine the imaging outcomes of emergency MRI as a first-line imaging method in patients with suspected CVST. In this retrospective cohort study, we analyzed emergency brain MRI referrals from a five-year period in a tertiary hospital for suspicion of CVST. We recorded patient characteristics, risk factors mentioned in the referrals, and imaging outcomes. Altogether 327 patients underwent emergency MRI on the grounds of suspected CVST. MRI showed evidence of CVST among five patients (1.5%). Imaging showed other clinically significant pathology in 15% of the patients and incidental findings in 5% of the patients. Despite clinical suspicion, the diagnostic yield of emergency MRI for CVST is low and similar to that previously reported for CT. MRI is an alternative imaging method devoid of ionizing radiation in patients with suspected CVST.

## 1. Background

Cerebral venous sinus thrombosis (CVST) is a potentially life-threatening neurological emergency condition defined as a blood clot in the major venous outlets of the brain [1]. CVST is a subset of cerebral venous thrombosis, referring to the presence of a blood clot, specifically in the dural venous sinuses. The incidence of CVST in the general population has previously been reported as 0.2 to 0.5 cases per 100,000 individuals per year, but a recent study has reported a higher incidence of 1.3 to 1.6 per 100,000 per year [2]. Yet CVST is much more often suspected in the emergency department on clinical grounds because its symptoms and risk factors are non-specific. A stereotypical patient is a young adult female with a history of smoking and hormonal oral contraceptive use, presenting with headaches [3,4].

Imaging options to rule out CVST are computed tomography (CT) and magnetic resonance imaging (MRI) [5,6,7,8,9]. A fairly recent meta-analysis from 2018 indicates that both CT and MRI have a high level of diagnostic accuracy in the differential diagnosis of venous thrombosis [10]. Non-enhanced CT and contrast-enhanced CT venography are widely available and cost-effective imaging options in an emergency setting, although they expose the patient to ionizing radiation.

Various MRI techniques, such as contrast-enhanced MRI and magnetic resonance venography (MRV), are also useful techniques for ruling out CVST. The major advantage of MRI over CT is that MRI offers better soft tissue discrimination and, thereby, higher sensitivity than CT for subtle brain pathology. MRI does not involve the use of ionizing radiation, which is beneficial in cases of pregnancy and young patients, although CT scans of the mother’s head cause minimal radiation dose for the fetus, much below the teratogenic threshold [11]. Disadvantages of MRI are its poorer availability in emergency departments and its higher cost. A previous study reported CT findings in patients with suspected CVST [5], but the additional value of MRI in terms of the prevalence of clinically significant findings in these patients is unknown.

The primary aim of the present study was to assess the imaging outcomes of emergency MRI among patients presented with clinically suspected CVST and to characterize these patients in terms of demographics, history, and specific signs and symptoms. We specifically focused on primary screening for CVST with MRI because of its high soft tissue contrast. We recorded other intracranial pathology encountered in emergency MRI among patients with a clinical suspicion of CVST to identify brain disorders that may clinically mimic CVST.

## 2. Materials and Methods

For this retrospective cohort study, we obtained permission from The Hospital District of Southwest Finland. A waiver for written patient consent was not sought from the institutional review board (IRB, called the Ethics Committee of The Hospital District of Southwest Finland) because it is not required by the national legislature for retrospective studies of existing data. The study was conducted in accordance with the Declaration of Helsinki. We first identified 8772 unique emergency brain MRI scans conducted between April 2014 and January 2019 from picture archiving and communication systems (PACS) and radiological information systems using standard brain MRI codes, carried out at an academic tertiary care referral center, performed on a Philips Ingenia 3 Tesla system. The MRI protocol included routine sequences such as T1- and T2-weighted imaging, fluid-attenuated inversion recovery (FLAIR), diffusion-weighted imaging (DWI), susceptibility-weighted imaging (SWI), 3D time-of-flight (TOF) arterial angiography, 2D TOF venography (MRV) (selected patients), and contrast-enhanced (CE) MRV (selected patients). Detailed MRI protocol used in the majority of the scans can be found in Supplemental Material. Imaging data were cross-referenced with those from electronic medical records (EMR).

To identify clinical suspicion of CVST, we queried referrals with the words “sinus thrombosis” and “(dural) venous sinus/es” and included patients in whom CVST was suspected either as a primary or secondary differential diagnosis. All consecutive patients between April 2014 and January 2019 were included regardless of the hour of examination. We excluded cases referred to MRI with previously diagnosed CVST at an outside institution or with another imaging modality, such as CT angiography. We recorded whether known risk factors for CVST were mentioned in the referrals and the pertinent findings in the MRI reports. Whether an MRI finding was considered potentially causally related to the symptoms and thus clinically significant was evaluated by a consensus procedure by a board-certified neurologist (P.Y.) and a neuroradiologist (M.N.). From the EMR, we recorded relevant clinical information, such as patient history, duration of headache, laboratory results, length of hospital stay, and final discharge diagnosis.

Results are typically expressed as percentages, medians, and interquartile ranges (IQR). We used Pearson T-tests and the Wilcoxon rank sum test to compare continuous variables and the Chi-square (*χ*^2^) test to compare ordinal data. The data were analyzed using JMP for Mac (Version 16.1 Pro. SAS Institute Inc., Cary, NC, USA, 1989–2019) and SAS version 9.4 (SAS Institute, Cary, NC, USA). *p*-values less than 0.05 were considered statistically significant.

## 3. Results

In total, 327 patients underwent emergency MRIs under clinical suspicion of CVST. Of these, 274 (84%) were female, and the median age was 30 (IQR: 23–38) years (Table 1). Medical risk factors for CVST were mentioned in 50% of the referrals, including smoking, oral contraception, pregnancy, puerperium, and thrombophilia. Twenty-three (7%) patients had two of these risk factors. The most prevalent clinical symptom at presentation was headache (91%), commonly accompanied by other symptoms such as nausea/vomiting or visual impairment. Nineteen patients (6%) had a previous diagnosis of a significant brain disease, such as brain tumor, hydrocephalus, Moyamoya disease, or previous cerebral infarction/hemorrhage. A total of 252 (77%) patients received contrast-enhanced MRV (T1 3D turbo-field echo), and 66 patients (20%) received 2D-TOF-MRV.

**Figure 1:**
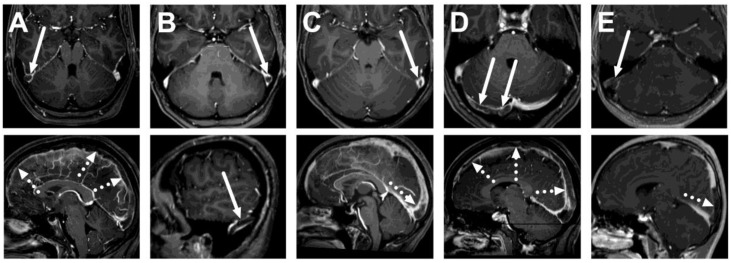
True positive cases of cerebral venous sinus thrombosis. Axial (top row) and sagittal (bottom row) images of post-gadolinium T1-weighted 3D MRI images of five patients: a 26-year-old female (**A**), a 33-year-old female (**B**), a 21-year-old female (**C**), a 27-year-old female (**D**), and a 16-year-old male (**E**). Thrombosis is seen as a hypointense filling defect against the high signal from the gadolinium-based contrast agent in the venous sinuses, usually in the transverse sinuses (arrows) and the superior sagittal sinus (dotted arrows).

**Figure 2:**
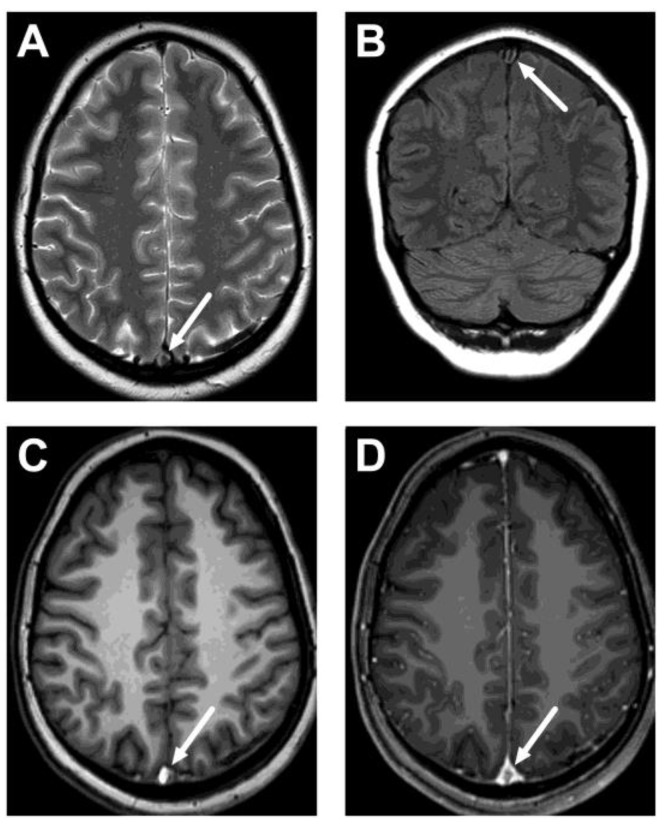
An example of cerebral venous sinus thrombosis on routine pre-contrast images. Images show a lack of normal flow void on T2-weighted (**A**) and fluid-attenuated inversion recovery (FLAIR) (**B**) images, as well as high signal on the pre-contrast T1-weighted image (**C**), in the superior sagittal sinus (arrows). Post-contrast T1-weighted image is provided for reference (**D**), showing the non-enhancing thrombus (arrow). Most patients did not show thrombosis on all routine sequences, however. The patient is the same as in Figure 1C.

Among the emergency MRI referrals to rule out CVST, we found five positive cases out of 327 (1.5%) (Figure 1). That is, 98.5% did not show CVST. All positive CVST findings were detected using contrast-enhanced MRV (5 out of 252 scans performed), whereas all 2D-TOF-MRV were unremarkable. Neither sequence showed any other pathology in the cerebral venous system. Positive cases were confirmed by follow-up MRI six months after the initial diagnosis, which showed partial or complete resolution of thrombosis. All but one of these patients were young females. All had thrombosis in the transverse sinus, and 4/5 had superior sagittal sinus involvement. These thrombi were variably shown by routine MRI sequences: on T2-weighted images in 1/5 patients, on FLAIR images in 2/5 patients, and on T1-weighted images in 4/5 patients (Figure 2, Table 2). Only one patient had parenchymal changes (edema, no hemorrhage).

Imaging was deemed completely unremarkable for 77%, whereas clinically significant intracranial pathology other than CVST was found in 48 (15%) cases: e.g., intracerebral hemorrhage, cerebral infarction, brain tumor, and sinusitis (Table 3). That is, 16% of patients had significant findings in MRI, including CVST. In addition, incidental findings were discovered in 17 (5%) cases, such as developmental venous anomalies and non-specific white matter lesions.

Male sex and high body mass index (BMI) were found to be statistically significantly associated with intracranial pathology in emergency MRI (Table 1). Pregnancy was found to decrease the risk of significant findings. Patient age, duration of headache before MRI, or presence of any of the individual symptoms had no statistically significant impact on whether or not significant findings were discovered.

Among patients in this study, only 61 (19%) underwent CT scanning before MRI, and 49 (80%) of these scans were considered unremarkable. That is, CT suggested significant pathology in six (10%) patients (ICH, infection), and CVST could not be definitively ruled out for the other six. The prevalence of significant findings in MRI was similar in patients with a previous unremarkable CT and in those who did not have CT at all (18% vs. 14%, *p* = 0.46, *χ*^2^ test).

The most prevalent discharge diagnosis was non-specified headache (30%), followed by migraine headache (21%) and tension headache (9%). The rest of the discharge diagnoses included a wide spectrum of different categories, including various types of nervous diseases, circulatory diseases, infections, and malignancies. Most (70%) patients were discharged from the hospital within the following 24 h.

## 4. Discussion

Diagnosing CVST is challenging, considering that the diagnostic yield of imaging for suspected CVST is relatively low in terms of how often it is suspected on clinical grounds. We found that emergency MRI showed signs of CVST in only 1.5% of all cases and other clinically significant intracranial pathology in 15% of cases. The most prevalent findings among these patients included sinusitis, intracerebral hemorrhage, cerebral infarction, and signs of intracranial hypertension. These novel results add to previous knowledge by showing that the rate of significant imaging outcomes of MRI does not significantly differ from that of CT in these patients.

Our findings regarding patient demographics, history, and specific signs and symptoms that have induced clinical suspicion for CVST are similar to those previously reported [12,13,14,15]. In our data, a typical patient was a young woman with a headache accompanied by nausea and focal neurological deficits, such as numbness or visual impairment. Imaging was deemed unremarkable for 84% of patients and with no CVST for 98% of patients. This demonstrates the high index of clinical suspicion and the low diagnostic yield that MRI seems to have in these patients.

Due to the small sample of patients with CVST, we did not attempt to create an algorithm to predict a positive imaging outcome based on risk factors, symptoms, and laboratory results. We did not consider it reasonable to draw further conclusions from these factors in this study.

We found that the yield of imaging suspected CVST cases using emergency MRI was greater among patients that were male or had high BMI. Females, especially those who were pregnant, underwent MRI more often with no detected intracranial pathology, which further underlines the high alert for suspicion of CVST in these patients. Patient age, duration of headache before MRI, or presence of individual symptoms had no impact on whether or not clinically significant findings were discovered in emergency MRI. From the clinical perspective, this indicates that medical imaging plays a considerable role in ruling out CVST regardless of these factors.

The prevalence of other significant findings in emergency MRI (15%) is only slightly higher than that previously reported for nonenhanced CT (11%) [5]. These relatively small differences in results might well be attributed to differences in patient management, demographical characteristics, or referral patterns between institutions. In retrospect, many of our significant findings could likely have been visible on CT (e.g., intracerebral hemorrhage, infarction), which in most circumstances might be sufficient in the emergency environment with shorter scan time and lower cost. On the other hand, MRI could show more subtle but potentially significant findings among these patients (e.g., small infarcts, signs of intracranial hypertension), but this ability of MRI did not translate into a considerably higher overall prevalence of significant pathology when compared with CT. In comparison to emergency MRI for non-traumatic headaches overall, the proportion of significant findings seems similar (16% vs. 20%) [16]. The small difference might be due to the younger age of patients in this study.

Dedicated emergency MRI is a feasible first-line imaging method for various acute pathology [17]. At our institution, this has become a routine modality, especially among young patients, because of its excellent soft tissue contrast and lack of ionizing radiation. Therefore, we sought to characterize MRI findings in suspected CVST in these patients in particular and not include patients who might have undergone only CT for similar clinical suspicion. We found that the prevalence of significant findings was similar in patients with previous unremarkable CT and in those who did not have CT at all. This analysis further confirms the role of MRI as a first-line imaging method in our emergency department for these patients. However, some sampling bias toward MRI cannot be excluded. Our patients were slightly younger than those previously reported as having undergone CT based on suspected CVST [5]. In addition, the availability of emergency MRI in our institution might lower the threshold to ask for imaging for young, mildly symptomatic patients compared to when only CT is available. Too liberal patient selection and possible overuse of MRI might partly explain low diagnostic yield, in addition to inherent difficulties in recognizing CVST because of non-specific symptoms and common risk factors.

Regarding imaging methodology, CE-MRV has the highest diagnostic accuracy in detecting CVST [18], whereas TOF-MRV has similar sensitivity but slightly lower specificity [19]. Routine MRI sequences have variable accuracies but generally perform less well than MRV methods [18,20]. At our institution, patients with suspected CVST receive MRV, mostly CE-MRV. We did not find any cases with isolated cortical venous thrombosis, which is a rare subtype of cerebral venous thrombosis, presenting with headaches, neurological deficits, and seizures [21,22]. Isolated cortical venous thrombosis would likely be visible using our protocol on SWI images [21] and on 3D-CE-MRV as well.

One of the major strengths of this study was the routine use of MRI in the emergency radiology department. Furthermore, this study reflects a true clinical situation due to its several patients with clinical suspicion of CVST at the same center over a period of almost five years, investigated under similar conditions. Due to the practice routine in the use of emergency MRI and a large sample size, these results are likely well generalizable to the whole population of patients with suspected CVST.

Yet, this study is limited by its retrospective, single-center design. In addition, guidelines for the clinical suspicion of CVST may vary from one hospital to another. Most importantly, emergency MRI is not routinely available in all institutions, limiting the generalizability of our findings. Conclusions are further limited by the fact that our observational study used varying MRI protocols, which may impact diagnostic accuracy. The choice between contrast-enhanced MRV and 2D-TOF-MRV is made by the on-call radiologist, and we could not examine the reasons for these choices in this observational retrospective study. We did not routinely use 3D phase-contrast MRV. MRI with MRV is a highly reliable method for diagnosing CVST, but conventional CT will remain the first imaging modality for most acute situations, simply due to wide-spread availability, fast scanning times, and ability to suggest most acute conditions, such as tumors, hemorrhages, and abscesses, although many of these conditions will eventually require MRI for an accurate diagnosis. Added value for MRI over CT as a primary screening tool stems mainly from its superior soft tissue discrimination and lack of ionizing radiation.

## 5. Conclusions

In conclusion, we found that the diagnostic yield of emergency imaging suspected CVST using MRI was low and similar to what has been reported for CT. In our data, significant findings were most likely found in patients that were male or had high BMI. Although imaging outcomes were close to similar to that reported for CT, the lack of ionizing radiation might favor the use of MRI as an alternative screening tool for intracranial pathology in emergency patients suspected of having CVST.

## Figures and Tables

**Table 1:** Clinical characteristics of patients with clinically suspected CVST who underwent emergency MRI.

	Clinically Significant MRI Finding ^1^ *N* = 53	No Significant MRI Finding *N* = 274	*p*-Value ^2^
**Sex**	***N* (%)**		
Female	39 (74)	235 (86)	0.027
Male	14 (26)	39 (14)	
**Age**	**Median (IQR)**		
Total (years)	28 (21–42)	30 (23–38)	0.957
Female (years)	29 (24–42)	29 (23–37)	0.585
Male (years)	24 (16–61)	33 (22–45)	0.545
**Body mass index**	**Data available (*N*): Median (IQR)**		
BMI (kg/m^2^)	41:28 (24–36)	181:25 (22–30)	0.018
**Medical risk factors**	***N* (%)**		
Smoking	14 (26)	54 (20)	0.271
Thrombophilia	1 (2)	6 (2)	0.889
Oral contraception	7 (13)	44 (16)	0.601
Pregnancy	1 (2)	39 (14)	0.012
Puerperium	0 (0)	11 (4)	0.138
**Symptoms**	***N* (%)**		
Headache	47 (89)	250 (91)	0.554
Nausea/vomiting	23 (43)	122 (45)	0.880
Visual impairment	17 (32)	91 (33)	0.872
Numbness	15 (28)	82 (30)	0.813
Vertigo	13 (25)	72 (26)	0.790
Photophobia	17 (32)	61 (22)	0.125
Neck muscle tension	14 (26)	57 (21)	0.364
Dysphasia	7 (13)	34 (12)	0.872
Seizure	2 (4)	6 (2)	0.495
No other symptoms than headache	2 (4)	24 (9)	0.219
**Imaging information**	***N* (%)**		
Contrast-enhanced MRI	48 (91)	204 (75)	0.011
**Additional clinical information**	**Median (IQR)**		
Duration of headache before MRI (days)	4 (2–10)	3 (1–10)	0.097
Length of hospital stay (days)	5 (0–9)	0 (0–0)	<0.001

MRI = Magnetic resonance imaging, CVST = Cerebral venous sinus thrombosis, IQR = Interquartile range. ^1^ Including CVST. ^2^ *p*-Values are associated with Chi-squared test for categorical variables and with Wilcoxon rank sum test for continuous variables.

**Table 2:** MRI findings in different sequences in the venous sinuses of patients with CVST and parenchymal changes (from all sequences). Patient codes correspond to those in Figure 1.

Patient	T2	FLAIR	SWI	T1 Pre-Contrast	T1 Post-Contrast	Parenchymal Change
**A**	Unremarkable	Loss of normal flow void	Mixed signal intensity	Hyperintense	Filling defect	None
**B**	Unremarkable	Unremarkable	Unremarkable	Unremarkable	Filling defect	None
**C**	Loss of normal flow void	Loss of normal flow void	Mixed signal intensity	Hyperintense	Filling defect	Edema
**D**	Unremarkable	Unremarkable	Unremarkable	Hyperintense	Filling defect	None
**E**	Unremarkable	Unremarkable	Unremarkable	Hyperintense	Filling defect	None

**Table 3:** Clinically significant MRI findings in patients with clinical suspicion of CVST.

MRI Finding	N (%)
Sinusitis *	10 (3.1)
Intracerebral hemorrhage	8 (2.4)
Cerebral infarction	7 (2.1)
Intracranial hypertension *	7 (2.1)
Cerebral venous sinus thrombosis	5 (1.5)
Blood vessel pathology	4 (1.2)
Brain tumor	4 (1.2)
Demyelination	3 (0.9)
Mastoiditis	2 (0.6)
Subarachnoid hemorrhage	1 (0.3)
Subdural hematoma	1 (0.3)
Arteriovenous Fistula	1 (0.3)
Intracranial Hypotension	1 (0.3)
**Total**	54 * (16)

* 1 patient presenting with both sinusitis and intracranial hypertension.

## Data Availability

Image data cannot be publicly shared because of the national legislature on patient data. Otherwise, all relevant data are within the manuscript and its supplementary information. Further inquiries should be addressed to J.H.

## Supplementary Materials

The following supporting information can be downloaded at: https://www.mdpi.com/article/10.3390/diagnostics13122052/s1, Detailed MRI protocol.
