# Supplementary material for: Imaging Outcomes of Emergency MRI in Patients with Suspected Cerebral Venous Sinus Thrombosis: A Retrospective Cohort Study
Source: Diagnostics (Basel). 2023 Jun 14;13(12):2052. doi: 10.3390/diagnostics13122052 (PMC10297083; doi:10.3390/diagnostics13122052)
Supplement: Supplementary file 1 [file diagnostics-13-02052-s001.zip › diagnostics-2416206-supplementary.pdf]

Supplementary Material

Detailed MRI protocol

Sequence	Orientation	Slice thickness	Parameters
T2	Axial	3 mm	TE 80 ms<br>TR 4300 ms
FLAIR	Coronal	4 mm	TE 125 ms<br>TR 11000 ms<br>TI 2800 ms
T1 3D TFE *	Axial	1 mm	TE 4 ms<br>TR 8.5 ms
DWI	Axial	4 mm	TE 87 ms<br>TR 4500 ms<br>b=1000 s/mm^2
SWI	Axial	2 mm	TE 28 ms<br>TR 20 ms
3D-TOF arterial angio	Axial	1.2 mm	TE 3.5 ms<br>TR 23 ms
2D-TOF venous angio **	Axial	3 mm	TE 3.3 ms<br>TR 19 ms

*Among 252 patients (77%), this sequence was repeated after intravenous gadolinium-based contrast agent (after which this sequence is referred to in the article as “CE-MRV”). **This sequence was performed among 66 patients (20%). DWI, diffusion-weighted imaging; FLAIR, fluid-attenuated inversion recovery; TE, time of echo; TFE, turbo field echo; TI, time of inversion; TOF, time-of-flight; TR, time of repetition; SWI, susceptibility-weighted imaging.
